# Strategies to reduce rates of severe endothermal heat-induced thrombosis following radiofrequency ablation

**DOI:** 10.1016/j.jvsv.2024.101864

**Published:** 2024-03-20

**Authors:** Baqir J. Kedwai, Joshua T. Geiger, Daniel J. Lehane, Roan J. Glocker, Karina A. Newhall, Grayson S. Pitcher, Jennifer L. Ellis, Adam J. Doyle

**Affiliations:** Division of Vascular Surgery, University of Rochester Medical Center, Rochester, NY

**Keywords:** Ablation, Anticoagulation, Thrombosis, Vein

## Abstract

**Objective:**

Endothermal heat-induced thrombosis (EHIT) is a potential complication of radiofrequency ablation (RFA). Data on effective prophylaxis of EHIT are limited. In 2018, a high-volume, single institution implemented strategies to decrease the incidence of EHIT, including a single periprocedural prophylactic dose of low-molecular-weight heparin to patients with a great saphenous vein (GSV) diameter of ≥8 mm or saphenofemoral junction (SFJ) diameter of ≥10 mm and limiting treatment to one vein per procedure. The size threshold was derived from existing literature. The study objective was to evaluate the effects of these institutional changes on thrombotic complication rates after RFA.

**Methods:**

A retrospective cohort control study was conducted using the Vascular Quality Initiative database. Data were collected for patients who underwent RFA with a GSV diameter of ≥8 mm or SFJ diameter of ≥10 mm from January 2015 to July 2022. The clinical end points were thrombotic complications (ie, thrombophlebitis, EHIT, deep vein thrombosis) and bleeding complications. Patient demographic and procedural variables were included in the analysis, and significant variables after univariable logistic regression were included in a multivariable logistic regression.

**Results:**

After the policy change, the overall vein center EHIT rate decreased from 2.6% to 1.5%, with a trend toward significance (*P* = .096). The inclusion criterion of a GSV diameter of ≥8 mm or an SFJ diameter of ≥10 mm yielded 845 patients, of whom 298 were treated before the policy change and 547 after. There was a significant reduction in the rate of EHIT classified as class ≥III (2.34 vs 0.366; *P* = .020) after the institutional changes. Treatment of two or more veins and an increased vein diameter were associated with an increased risk of EHIT (*P* = .049 and *P* < .001, respectively). No significant association was found between periprocedural anticoagulation and all-cause thrombotic complications or EHIT (*P* = .563 and *P* = .885, respectively).

**Conclusions:**

The institutional policy changes have led to lower rates of EHIT, with a reduction in severe EHIT rates in patients with an ≥8-mm diameter GSV or a ≥10-mm diameter SFJ treated with RFA. Of the changes implemented, restricting treatment to one vein was associated with a reduction in severe EHIT. No association was found with periprocedural low-molecular-weight heparin, although a type 2 error might have occurred. Alternative strategies to prevent thrombotic complications should be explored, such as increasing the dosage and duration of periprocedural anticoagulation, antiplatelet use, and nonpharmacologic strategies.

**Article Highlights**

• **Type of Research:** A single-center, retrospective cohort study
• **Key Findings:** In a study of 845 patients at a high-volume vein center, there was a significant decrease in severe endothermal heat-induced thrombosis (EHIT) rates from 2.34% to 0.37% after institutional changes to the radiofrequency ablation protocol. Restricting treatment to one vein was associated with this reduction in severe EHIT.
• **Take Home Message:** Institutional modifications in radiofrequency ablation practices at a high-volume vein center have led to a significant reduction in severe EHIT, conferring a beneficial impact on patient outcomes.

Chronic venous insufficiency (CVI) is estimated to affect 25 million adults in the United States.^1^ In 80% of cases, the etiology of CVI is superficial venous reflux and venous hypertension secondary to valvular incompetence of the great saphenous vein (GSV).^2^^,^^3^ The clinical spectrum of this disease ranges from hyperpigmentation and eczema of the lower extremities to wounds and ulceration, with significant effects on patient quality of life and healthcare resources.^1^^,^^4^, ^5^, ^6^ For patients with an inadequate response to conservative management, minimally invasive venous ablative therapy is most commonly performed using either radiofrequency ablation (RFA) or endothermal laser ablation.^7^, ^8^, ^9^

Ablation therapies have high rates of technical success and are associated with long-term improvements in symptoms and patient quality of life.^10^, ^11^, ^12^ Endothermal heat-induced thrombosis (EHIT) is a well-documented short-term complication of ablation therapies that occurs when thrombus from the treated vein segment propagates to the deep vein system, placing patients at risk of deep vein thrombosis (DVT) and pulmonary embolism.^13^, ^14^, ^15^, ^16^, ^17^, ^18^ Kabnick et al^15^ previously developed a four-tiered classification system to guide the diagnosis and management of EHIT. However, data regarding effective prophylaxis and institutional protocols to mitigate postablation thrombotic complications are limited.

In 2018, the University of Rochester Vein Center implemented a series of strategies to minimize the occurrence of EHIT and related thrombotic complications. These strategies included administration of a single preoperative prophylactic dose of low-molecular-weight heparin (LMWH) to patients with a great saphenous vein (GSV) diameter of ≥8 mm or saphenofemoral junction (SFJ) diameter of ≥10 mm and limiting RFA treatment to one vein per procedure. The vein diameter thresholds were derived from a preliminary analysis of institutional data for an internal quality improvement initiative and supported by multiple studies examining the risk factors for EHIT.^19^^,^^20^

The anticoagulation protocol involved administration of one 40-mg dose of subcutaneous enoxaparin sodium (Lovenox; Sanofi) immediately before the scheduled procedure for all patients, except for those already receiving a chronic anticoagulation regimen. Patients receiving chronic anticoagulation continued their prescribed regimen. The study was limited to RFA because the providers at the Vein Center do not routinely perform laser ablation. The objective of this study was to evaluate the impact of these institutional modifications on the rates of thrombotic and bleeding complications after RFA.

## Methods

A retrospective cohort study was conducted for all patients undergoing RFA with a GSV or an anterior accessory GSV (AAGSV) diameter of ≥8 mm or SFJ diameter of ≥10 mm at the University of Rochester Vein Center. All patients who underwent treatment of superficial venous reflux between January 2015 and July 2022 were identified through the prospectively maintained institutional Vascular Quality Initiative database. Patients aged >18 years, treated with RFA, and meeting the vein size criteria were included in the study. Patients treated with endovenous laser ablation, stab phlebectomy, ligation, stripping, or surgery and patients who did not meet the vein size criteria were excluded.

Clinical data were obtained from the prospective database and supplemented through a review of the patients' electronic medical records. The data collected included patient demographics, comorbidities, medication use, operative details, perioperative outcomes, and short- and long-term outcomes. The University of Rochester's research subjects review board approved the protocol of this study and waived the requirement for patient consent.

A pre–post analysis was conducted comparing the clinical outcomes of patients before and after the institutional practice changes in February 2018. The primary outcome was the incidence of EHIT, severe EHIT, and all-cause thrombotic complications in patients with a GSV or an AAGSV diameter of ≥8 mm or an SFJ diameter of ≥10 mm. Severe EHIT was defined as class ≥III, as described by the American Venous Forum EHIT Classification System (Supplementary Table I, online only).^15^ In accordance with the guidelines, class III EHIT was defined as propagated thrombus comprising >50% of the adjacent deep vein lumen, and class IV was defined as propagated thrombus resulting in occlusion of an adjacent deep vein.^15^ All-cause thrombotic complications was a composite variable consisting of EHIT, DVT, and superficial thrombophlebitis incidence. All patients were screened for thrombotic complications with routine Doppler ultrasound of the treated extremity at 1 week of follow-up. The secondary outcomes analyzed were bleeding complications, defined as the development of a hematoma or other bleeding events requiring intervention, and the rates of recanalization.

The Student *t* test and Pearson χ^2^ test were used to evaluate for differences between the treatment cohorts. The Youden J statistic was used for cut-point analyses. An α of 0.05 was the threshold to evaluate for statistical significance in all analyses. Univariate logistic regression was used to identify statistically significant associations between patient and procedural variables and the primary and secondary outcomes. Variables yielding *P* < .05 on univariate analysis were included in a multivariable logistic regression model, followed by backward elimination using a threshold of *P* < .05.

Data extraction and cleaning was done using SAS, version 9.2 (SAS Institute) and R statistical software (R version 2022.12.0+353; R Foundation for Statistical 136 Computing). Statistical analyses were conducted using R statistical software.

## Results

**Fig 1:**
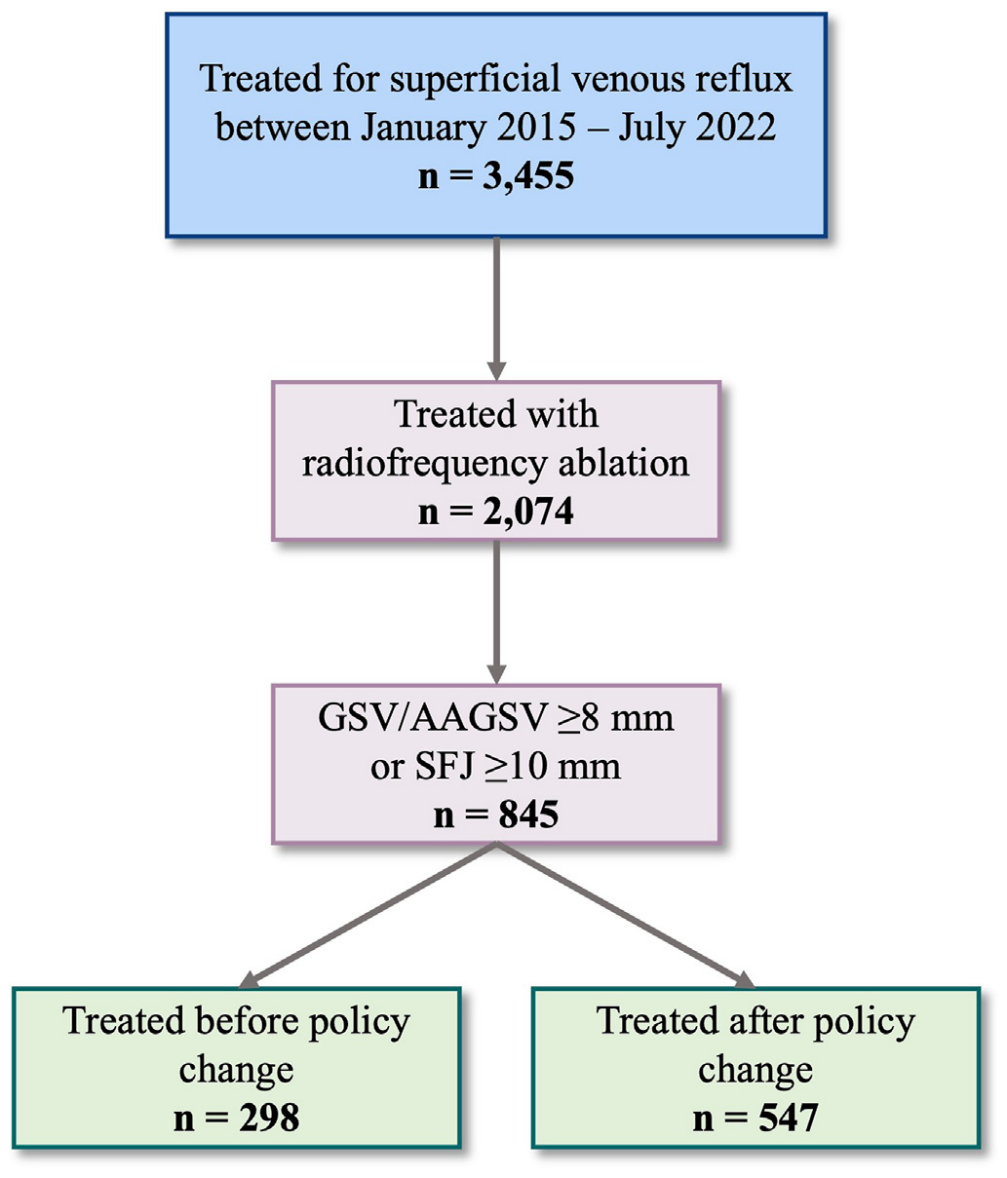
Flowchart of cohort selection. *AAGSV*, Anterior accessory great saphenous vein; *GSV*, great saphenous vein.

A total of 3455 patients were treated for superficial venous reflux disease between January 2015 and July 2022 and had 30-day follow-up data available. A portion of these patients were treated for bilateral disease, such that 4026 unique limbs were treated during this time. Our inclusion and exclusion criteria yielded 845 patients (887 unique limbs), of whom 298 patients were treated before the policy change and 547 after (Fig 1).

The two cohorts were similar with respect to age, body mass index (BMI), gender, and diameter of the vein treated. The post-policy change cohort had more patients receiving periprocedural LMWH and fewer patients treated for two or more veins, as dictated by the institutional policy change. Additionally, the post-policy change cohort had longer vein segments treated and a higher preoperative venous clinical severity score. The patient characteristics of both cohorts are summarized in Table I.

**Table I:** Demographic and operative characteristics

	After intervention		*P* value
	No (n = 298)	Yes (n = 547)	
Age, years	54.9 ± 13.0	56.0 ± 13.2	.236
BMI, kg/m^2^	32.3 ± 7.47	32.7 ± 8.57	.476
Female gender	181 (60.7)	334 (61.1)	.927
White race	280 (94.0)	491 (89.8)	.041
Black race	8 (2.68)	25 (4.57)	.181
Hispanic ethnicity	11 (3.69)	22 (4.02)	.813
Chronic anticoagulation	9 (3.02)	55 (10.1)	<.001
Platelet count, 1000/μL	227 ± 48.5	253 ± 46.2	.012
Periprocedural antiplatelet therapy	4 (1.34)	10 (1.83)	.598
Periprocedural LMWH	1 (0.34)	440 (80.4)	<.001
Multiple veins treated	142 (47.7)	8 (1.46)	<.001
Maximum vein length, cm	46.2 ± 13.5	48.7 ± 16.0	.020
Maximum vein diameter, mm	9.96 ± 2.35	10.0 ± 2.13	.804
Maximum preoperative VCSS	6.74 ± 2.75	7.89 ± 3.41	<.001

*BMI*, Body mass index; *LMWH*, low-molecular-weight heparin; *VCSS*, venous clinical severity score.

Data presented as mean ± standard deviation or number (%).

After the institutional policy change in February 2018, a decrease occurred in the rates of all-cause thrombotic complications (4.02% vs 2.97%; *P* = .211), EHIT (2.6% vs 1.5%; *P* = .096), and severe EHIT (1.15% vs 0.29%; *P* = .027) for all patients treated with RFA in the Vein Center, including patients who met the size criteria for anticoagulation and those who did not.

**Fig 2:**
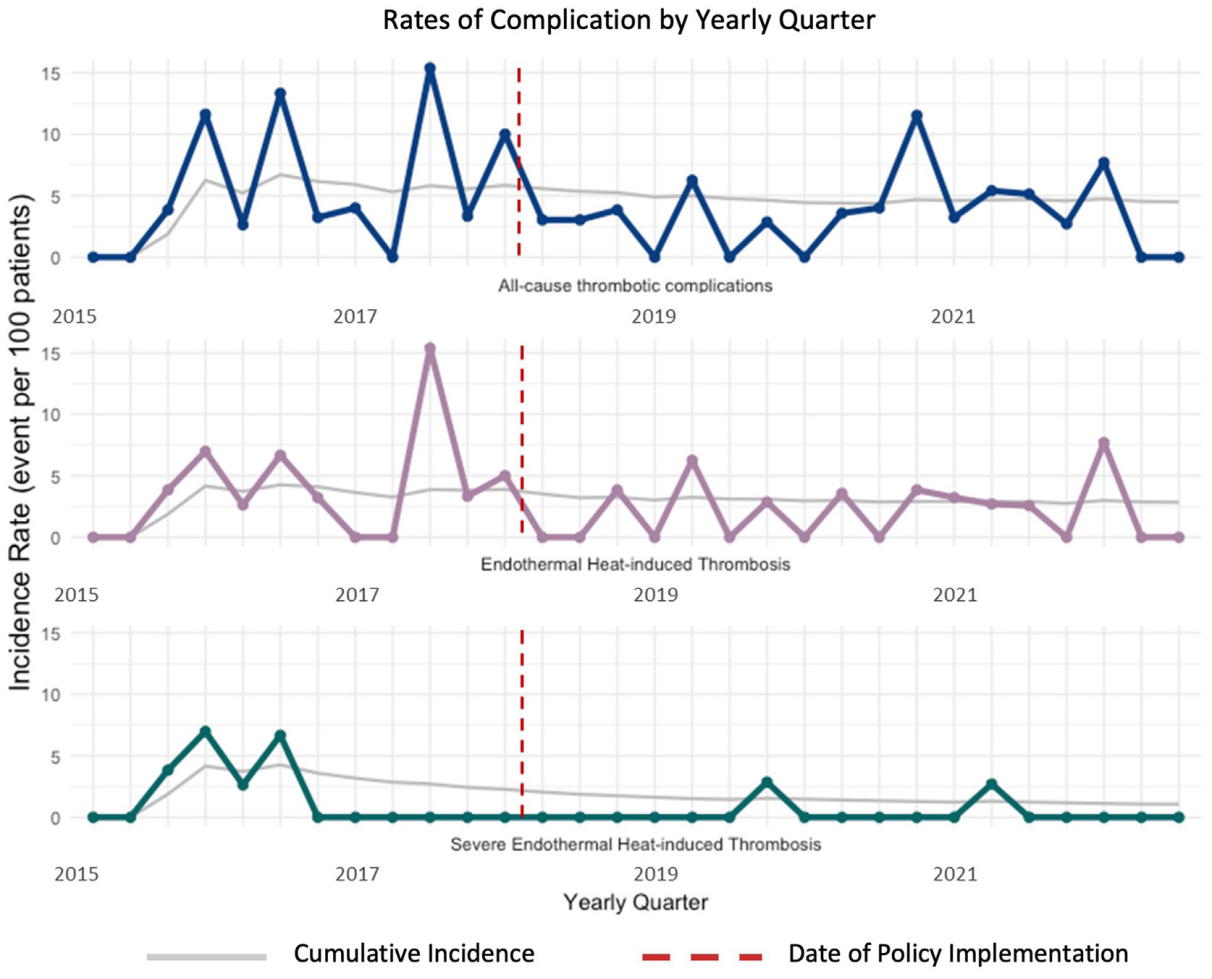
Run chart depicting incidence rate of all-cause thrombotic complications, endothermal heat-induced thrombosis (EHIT), and severe EHIT stratified by yearly quarter.

The trends in thrombotic complications, EHIT rates, and severe EHIT rates observed across all RFA procedures were similar in the cohort of patients with a GSV or an AAGSV diameter of ≥8 mm or an SFJ diameter of ≥10 mm who received a perioperative dose of LMWH. In this cohort, the rate of severe EHIT after RFA decreased from 2.34% to 0.66% (*P* = .020) after the policy change. No increase occurred in bleeding complications (0.00% before and after the policy change) or recanalization (0.336% vs 0.731%; *P* = .663) rates in the patients who received anticoagulation. The key clinical outcomes for patients eligible for receiving perioperative LMWH are summarized in Table II. A run chart illustrating the incidence rate of all-cause thrombotic complications, EHIT, and severe EHIT by yearly quarter from January 2015 to July 2022 is depicted in Fig 2.

**Table II:** Patient outcomes

Variable	After intervention		*P* value
	No (n = 298)	Yes (n = 547)	
All-cause thrombotic complications	17 (5.70)	21 (3.84)	.214
EHIT	11 (3.69)	13 (2.38)	.275
EHIT class ≥III	7 (2.34)	2 (0.366)	.020
Bleeding complications	0 (0.00)	0 (0.00)	1.000
Recanalization	1 (0.336)	4 (0.731)	.663

*EHIT*, Endothermal heat-induced thrombosis.

Data presented as number (%).

The rates of complications during the study period for a subgroup of patients with GSV and SFJ diameters less than the size threshold of 8 mm and 10 mm, respectively, are summarized in Supplementary Table II (online only). This group of patients did not receive anticoagulation because they did not meet the size criteria but were still limited to treatment of one vein per procedure. Consistent with the overall Vein Center rates, a decrease occurred in thrombotic complication rates from 2.76% to 2.41%, although the difference was not significant (*P* = .701). In this subgroup, no significant change was found in the rates of severe EHIT during the study period (0.251% vs 0.241%; *P* = .270).

Before the practice change, multiple vein treatments were routinely conducted. The target veins could be on either ipsilateral or contralateral legs. Treatment of multiple veins on one leg had similar rates of thrombotic complications compared with treatment of both legs. The various vein combinations treated before the practice change and the complication rates associated with treating multiple veins on one leg vs both legs are summarized in Supplementary Table III (online only). Logistic regression analyses of the demographic, comorbidity, and operative variables found the maximum vein diameter to be predictive of all-cause thrombotic complications (average maximum diameter of 9.9 mm in the cohort without complications vs 11.4 mm in the cohort with complications; *P* = .045; odds ratio [OR], 1.201), EHIT (9.9 mm vs 12.2 mm; *P* < .001; OR, 1.284), and severe EHIT (10.0 mm vs 13.6 mm; *P* < .001; OR, 1.367). The treatment of multiple veins was found to be predictive of both EHIT (17.3% of those without complications vs 33.3% of those with complications; *P* = .040; OR, 2.540) and severe EHIT (17.5% vs 44.4%; *P* = .036; OR, 4.434). Chronic or periprocedural anticoagulation with LMWH, unfractionated heparin, or other medications was not positively or negatively predictive of complications. The BMI was found to be protective of all-cause thrombotic complications. The variables predictive of primary and secondary outcomes are detailed in Table III.

**Table III:** Univariate and multivariable regression results for significant predictive variables for all-cause thrombosis, endothermal heat-induced thrombosis (EHIT), and severe EHIT

Variable	Complication		*P* value		OR (95% CI)
	No	Yes	Univariate	Multivariable	
All-cause thrombosis					
BMI, kg/m^2^	32.7 ± 8.26	29.7 ± 6.34	.030	.045	0.956 (0.914-0.998)
Maximum diameter, mm	9.92 ± 2.11	11.4 ± 3.56	< .001	< .001	1.201 (1.080-1.329)
EHIT					
Multiple veins treated	142 (17.3)	8 (33.3)	.049	.040	2.540 (0.995-6.039)
Maximum diameter, mm	9.92 ± 2.10	12.2 ± 4.16	< .001	< .001	1.284 (1.143-1.441)
Severe EHIT					
Multiple veins treated	146 (17.5)	4 (44.4)	.049	.036	4.434 (1.038-18.418)
Maximum diameter, mm	10.0 ± 2.12	13.6 ± 5.68	< .001	< .001	1.367 (1.168-1.600)

*BMI*, Body mass index; *CI*, confidence interval; *OR*, odds ratio.

Data presented as mean ± standard deviation or number (%), unless noted otherwise.

A cut-point analysis was conducted to assess the sensitivity and specificity of various vein diameters for thrombotic and bleeding complications. The Youden index and vein diameters of 8 mm, 9 mm, and 10 mm were tested. The Youden index identified a vein diameter of 9.3 mm as optimal for the detection of all-cause thrombotic complications and 9.5 mm for the detection of EHIT and severe EHIT. The results of the cut-point analyses are summarized in Table IV.

**Table IV:** Sensitivity and specificity analysis results of cut-points for optimal detection of thrombotic and bleeding complications

	Youden index		8 mm		9 mm		10 mm	
	Sensitivity	Specificity	Sensitivity	Specificity	Sensitivity	Specificity	Sensitivity	Specificity
Thrombotic complication	0.391	0.810	0.551	0.597	0.464	0.746	0.377	0.838
Bleeding complication	0.635	1.000	0.408	1.000	0.261	1.000	0.170	1.000
EHIT	0.462	0.823	0.615	0.596	0.564	0.745	0.462	0.836
Severe EHIT	0.667	0.813	0.750	0.594	0.750	0.742	0.667	0.834

*EHIT*, Endothermal heat-induced thrombosis.

## Discussion

Although infrequent, EHIT is a complication unique to thermal ablation that exposes patients to an increased risk of DVT and pulmonary embolism.^14^^,^^15^^,^^18^ Since 2018, the University of Rochester Vein Center has administered a periprocedural prophylactic dose of LMWH to patients with large GSV or SFJ diameters being treated with RFA and has limited the number of veins treated per procedure to one. The objective of the present study was to assess the impact of these institutional practice changes on the incidence of EHIT and related thrombotic complications after RFA.

The pre- and post-policy change cohorts were similar with respect to age, BMI, gender, and vein diameter. Several factors differed between the two cohorts including race, chronic anticoagulation use, vein length, platelet count, and preoperative venous clinical severity score. Differences in these factors, and additional unaccounted for variables, are inherent to the retrospective design of the study but are important to consider as potential confounders when evaluating the presented results.

After implementation of the thromboprophylaxis protocols, there was a statistically significant decrease in the rates of severe EHIT after RFA procedures, with an associated decrease in the rates of all-cause thrombotic complications and EHIT that were not significant. Although practice guidelines recommend against treatment of class I and class II EHIT in average-risk patients, there is strong evidence in support of regular surveillance and therapeutic anticoagulation therapy for patients with severe EHIT.

At our institution, all patients were screened for thrombosis at the 1-week follow-up with Doppler ultrasound. Patients with severe EHIT or DVT events identified through screening were treated with 100 mg of enoxaparin sodium (Lovenox) twice daily for 7 days and transitioned to oral anticoagulation, most commonly rivaroxaban (Xarelto; Janssen), if thrombus persisted. Therefore, the reduction in severe EHIT represents a clinically meaningful change that reduced patients' exposure to therapeutic levels of anticoagulation and additional clinical monitoring despite no significant decrease in overall EHIT rates. This additional benefit was obtained without an increase in bleeding complications secondary to the prophylactic dose of LMWH.

Our vein center successfully instituted the practice change of limiting RFA treatment to one vein per procedure. Multiple veins were treated in 47.7% of cases before the policy change but only 1.46% of cases after the policy change (Table I). Limiting the number of veins treated reduced the number of thermomechanical insults to a patient's vasculature, reducing the risk of thrombus formation. It also reduced the operative time, a factor previously associated with the risk of EHIT formation.^17^ Regression analyses found multiple vein treatments to be predictive of EHIT and severe EHIT, supporting our practice change as an effective preventative measure. Of the 1144 patients with venous reflux in multiple veins treated after the policy change, 356 patients returned for treatment of another vein. Limiting the treatments of these patients to one vein per procedure was likely the primary cause of the overall trend toward decreasing RFA-related thrombotic complications in the Vein Center. Further supporting this is the cohort of patients who did not meet the size criteria for periprocedural anticoagulation (Supplementary Table II, online only), because they also experienced decreased rates of all-cause thrombosis during the study period.

The use of perioperative LMWH was not predictive of a lower rate of thrombotic complications for patients with a large vein diameter in our regression analyses. In contrast, the vein diameter was a significant predictor of all-cause thrombotic complications, EHIT, and severe EHIT, as previously reported in the literature.^21^, ^22^, ^23^, ^24^ Our selection criteria for periprocedural anticoagulation was supported by studies that found a GSV of ≥8 mm and an SFJ of ≥10 mm to be associated with higher rates of EHIT.^13^^,^^20^ However, the average maximum vein diameter in patients with thrombotic complications in our study was greater than the size threshold used for anticoagulation. A cut-point analysis was conducted to identify the vein diameter that would optimize the sensitivity and specificity for thrombotic events. Using the Youden index method, the optimal cutoff value was identified to be 9.3 mm for all-cause thrombotic complications and 9.5 mm for EHIT (Table IV). Therefore, it is possible that no significant benefit from anticoagulation was observed because the threshold for anticoagulation was lower than the thresholds of 9.3 mm and 9.5 mm.

Our results indicate prevention of approximately two severe EHIT events per 100 patients treated. Prophylactic anticoagulation and antiplatelet medications have been shown to reduce the rates of venous thromboembolism; however, no consensus has been reached regarding the ideal agent, dose, or scheduling.^25^, ^26^, ^27^, ^28^ As such, the prophylactic regimen in the present study might provide inadequate protection against thrombotic events. This lack of observed effect might be compounded by the small sample size of the study. Assuming a 2% decrease in EHIT incidence from anticoagulation, a sample size of 1158 patients would be needed to adequately power the study. Thus, the lack of an association between anticoagulation and thrombotic complications might be the result of a type 2 error.

There is currently no consensus on the appropriate preventative strategies to reduce the rate of EHIT incidence. Our institution's practice changes have led to a significant reduction in severe EHIT occurrence, with a trend toward a reduction in all-cause thrombotic complications and EHIT and no increase in bleeding complications. Future studies should examine a larger cohort of patients, study the effect of varied perioperative anticoagulation and antiplatelet protocols, and explore additional nonpharmacologic strategies to reduce risk of EHIT.

### Study limitations

This work has some limitations that should be considered when interpreting its findings. Our study relies on retrospective data, which introduces a potential selection bias and confounding variables. Furthermore, differences in patient characteristics between the pre- and post-policy cohorts could also confound the outcomes. The small sample size could have limited the statistical power to detect significant differences in thrombotic complications, and a single dose of periprocedural LMWH might not be a sufficient regimen to maximally affect thrombotic complications. The research is limited to a single institution, potentially limiting its generalizability, and further multicenter studies are needed to provide a more comprehensive understanding of the institutional practice changes' effectiveness.

## Conclusions

The present study offers valuable insights into the impact of institutional modifications on thrombotic and bleeding complications after RFA for superficial venous reflux at a high-volume vein center. Since implementing the changes, there has been a significant decrease in severe EHIT, with a consistent trend toward lower rates of all-cause thrombotic complications and EHIT. These findings suggest that the institutional practice changes have had a clinically meaningful and beneficial impact on patient outcomes. Further studies with larger cohorts are warranted to confirm these positive outcomes, because they hold promise for improving CVI treatment strategies and enhancing patient safety.

## Author contributions

Conception and design: BK, JG, AD

Analysis and interpretation: BK, JG, DL, RG, KN, GP, JE, AD

Data collection: BK, JG

Writing the article: BK, JG

Critical revision of the article: BK, JG, DL, RG, KN, GP, JE, AD

Final approval of the article: BK, JG, DL, RG, KN, GP, JE, AD

Statistical analysis: BK, JG

Obtained funding: Not applicable

Overall responsibility: AD

BK and JG contributed equally to this article and share co-first authorship.

## Disclosures

None.

## Figures and Tables

**Supplementary Table I (online only):** Classification and treatment of endothermal heat-induced thrombosis (EHIT) per recommendations from American Venous Forum and Society for Vascular Surgery

Class	Definition	Treatment recommendation	Strength of recommendation and level of evidence
I	Thrombus without propagation into deep vein: a, peripheral to superficial epigastric vein; b, central to superficial epigastric vein, up to and including deep vein junction	No treatment or surveillance	2C
II	Thrombus propagation into adjacent deep vein but comprising <50% of deep vein lumen	No treatment, weekly surveillance until thrombus resolution; for high-risk patients, consider antiplatelet therapy vs anticoagulation; discontinue treatment following thrombus retraction or resolution	2C
III	Thrombus propagation into adjacent deep vein but comprising >50% of deep vein lumen	Therapeutic anticoagulation, weekly surveillance; discontinue treatment following thrombus retraction or resolution	1B
IV	Occlusive deep vein thrombosis contiguous with treated superficial vein	Treatment should be individualized, considering risks and benefits to patient; reference can be made to CHEST guidelines for treatment of deep vein thrombosis	1A

Adapted from Kabnick et al^15^ (recommendations from the American Venous Forum and the Society for Vascular Surgery).

**Supplementary Table II (online only):** Radiofrequency ablation (RFA) outcomes for patients with great saphenous vein (GSV) or anterior accessory GSV (AAGSV) diameter <8 mm or saphenofemoral junction (SFJ) diameter <10 mm

Variable	After intervention		*P* value
	No (n = 398)	Yes (n = 830)	
All-cause thrombotic complications	11 (2.76)	20 (2.41)	.701
EHIT	7 (1.76)	8 (0.964)	.270
EHIT class ≥III	1 (0.251)	2 (0.241)	1.000
Bleeding complications	1 (0.251)	1 (0.120)	.543
Recanalization	2 (0.503)	1 (0.120)	.254

*EHIT*, Endothermal heat-induced thrombosis.

Data presented as number (%).

**Supplementary Table III (online only):** Comparison of vessel targets and complication rates for multiple vein treatments on one leg vs multiple vein treatments on two legs

Variable	One leg (n = 96)	Two legs (n = 211)
Vein combination		
AAGSV thigh–AAGSV thigh	0 (0.00)	6 (2.84)
AAGSV thigh–GSV calf	8 (8.33)	4 (1.90)
AAGSV thigh–GSV thigh	35 (36.5)	13 (6.16)
AAGSV thigh–GSV thigh/calf	0 (0.00)	1 (0.474)
AAGSV thigh–SSV calf	5 (5.21)	2 (0.948)
GSV calf–SAGSV thigh	1 (1.04)	0 (0.00)
GSV thigh/calf–AAGSV calf	1 (1.04)	0 (0.00)
GSV thigh/calf–GSV thigh/calf	0 (0.00)	5 (2.37)
GSV thigh/calf–GSV thigh	0 (0.00)	1 (0.474)
GSV thigh/calf–SSV calf	1 (1.04)	1 (0.474)
GSV thigh–GSV calf	2 (2.08)	4 (1.90)
GSV thigh–GSV thigh	2 (2.08)	162 (76.8)
GSV thigh–SAGSV thigh	0 (0.00)	1 (0.474)
GSV thigh–SSV calf	39 (40.1)	10 (4.74)
GSV thigh–SSV thigh	1 (1.04)	0 (0.00)
SAGSV thigh–SSV calf	1 (1.04)	0 (0.00)
SAGSV thigh–SAGSV thigh	0 (0.00)	1 (0.474)
All-cause thrombotic complications	4 (4.12)	11 (5.21)
EHIT	4 (4.12)	7 (3.32)
EHIT class ≥III	1 (1.04)	3 (1.42)
Bleeding complications	0 (0.00)	1 (0.474)

*AAGSV*, Anterior accessory great saphenous vein; *EHIT*, endothermal heat-induced thrombosis; *GSV*, great saphenous vein; *SAGSV*, superficial accessory great saphenous vein; *SSV*, small saphenous vein.

Data presented as number (%).
